# Iatrogenic Vaginal Wall Perforation during Cesarean Section

**DOI:** 10.31662/jmaj.2021-0228

**Published:** 2022-03-18

**Authors:** Asako Watanabe, Shunji Suzuki

**Affiliations:** 1 Department of Obstetrics and Gynecology, Nippon Medical School, Tokyo, Japan

**Keywords:** iatrogenic vaginal wall perforation, cesarean section, surgical complication

## Abstract

We present a case of vaginal wall perforation during cesarean section. The cause of the perforation remained unclear. Although such conditions are rare, it is necessary to carefully check the condition of the vagina after performing cesarean section.

## Introduction

In cases of forced delivery or forceps/vacuum-extraction, a large amount of force is rapidly applied to the vaginal wall and perineum, causing damage or tears in the vaginal wall, sphincter, or rectal wall ^(1)^. For example, in approximately 85% of vaginal births, the parturients undergo perineal lacerations and/or episiotomy ^(2)^. However, we present a case of vaginal wall perforation during cesarean section.

## Case Report

A 27-year-old nulliparous female patient was referred to our institute because of premature rupture of the membranes at 30 weeks of gestation. Her temperature was 37.3°C, and there were no clinically discernible findings of chorioamnionitis; however, blood test findings included a white blood cell count of 12,700/μL and C-reactive protein level of 6.7 mg/dL. Emergent cesarean section was performed due to intrauterine infection. On internal examination just before the cesarean section, the dilatation of the uterine ostium was 4 cm, and the fetal head had descended to station ±0. During the cesarean section, however, the fetal head was descended into the pelvis, complicating the delivery. During the surgery, we did not perform transvaginal examination, such as lifting the fetal head during the cesarean section. The surgeon managed to lift the fetal head and delivered it out of the abdominal cavity. A 1,397-g, healthy female infant was delivered with Apgar scores of 8 and 9 at 1 and 5 minutes, respectively. The umbilical artery pH was 7.402. The total blood loss during cesarean section was 480 g. Postoperative speculum showed no abnormalities in the vagina; however, a histopathological examination revealed acute chorioamnionitis, which is the inflammation of the fetal membranes amnion and chorion, and funisitis, which is the infiltration of fetal neutrophils from the umbilical vessels into Wharton’s jelly.

**Figure 1.**
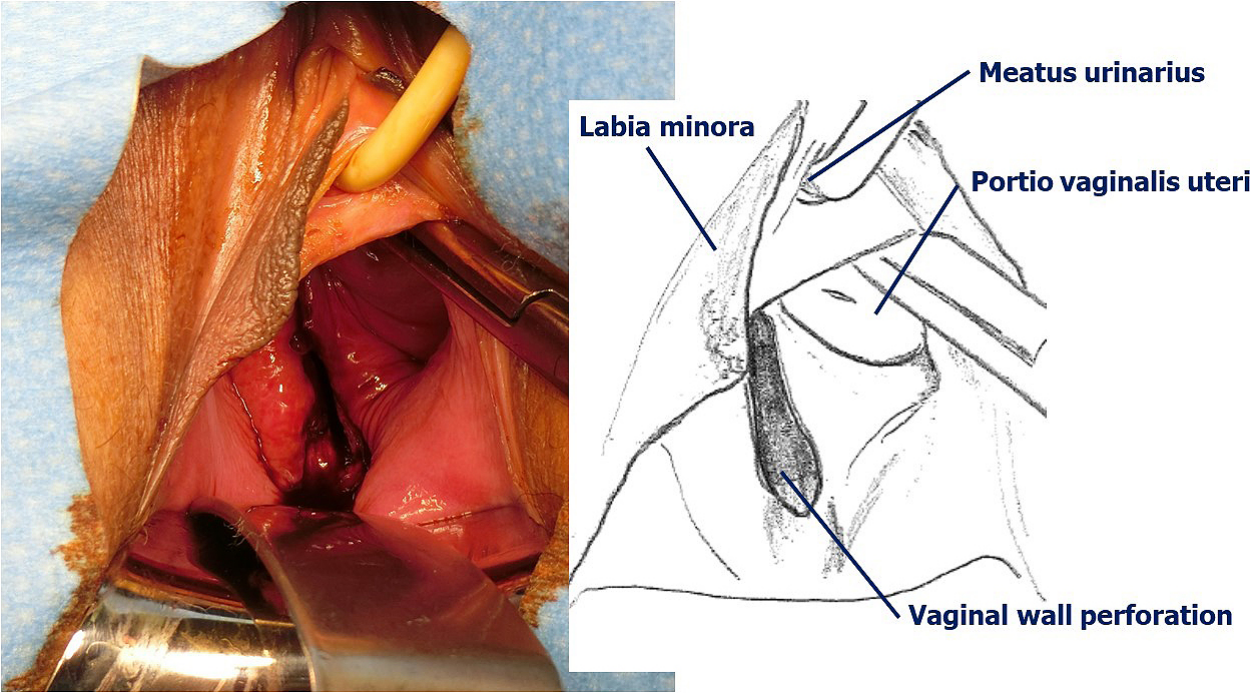
Perforation of posterior lateral vaginal wall.

**Figure 2.**
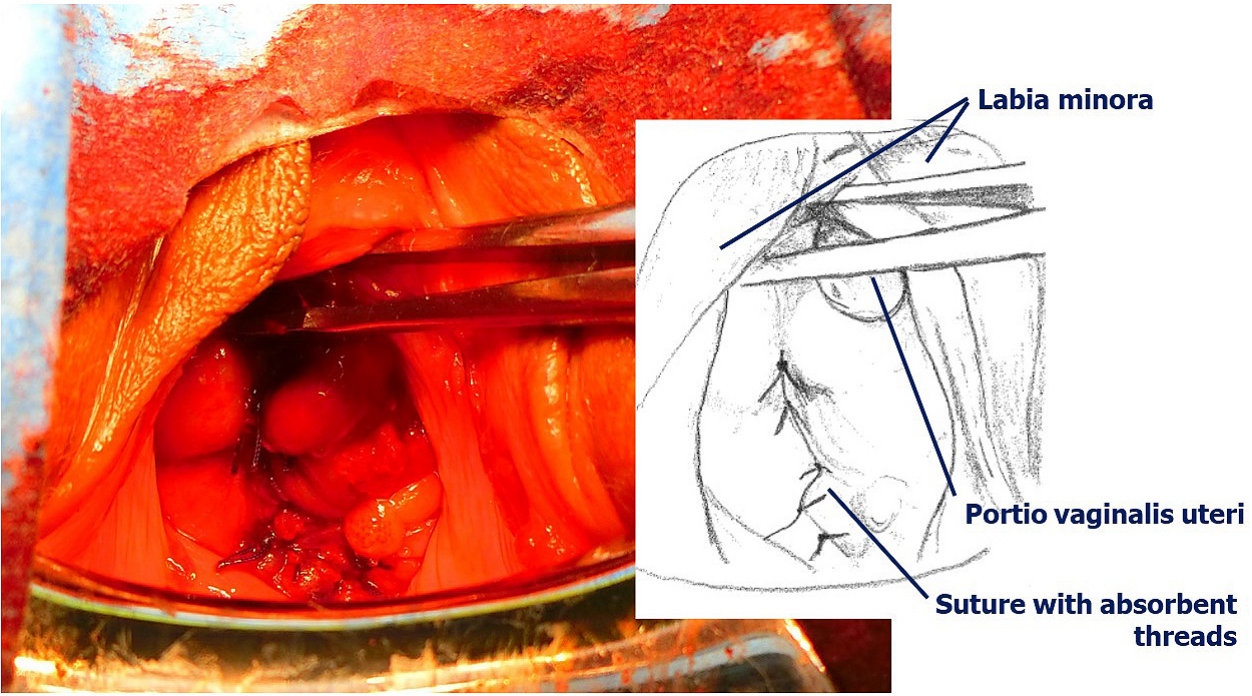
Vaginal wall sutured with absorbent threads.

At the routine examination on day 2 after surgery, the perforation of the posterior lateral vaginal wall with prolapse of the ileum requiring suture was observed (Figure 1). The peritoneum and vaginal wall were sutured separately with absorbent threads (Figure 2), and the patient was discharged 1 week later.

**Figure 3.**
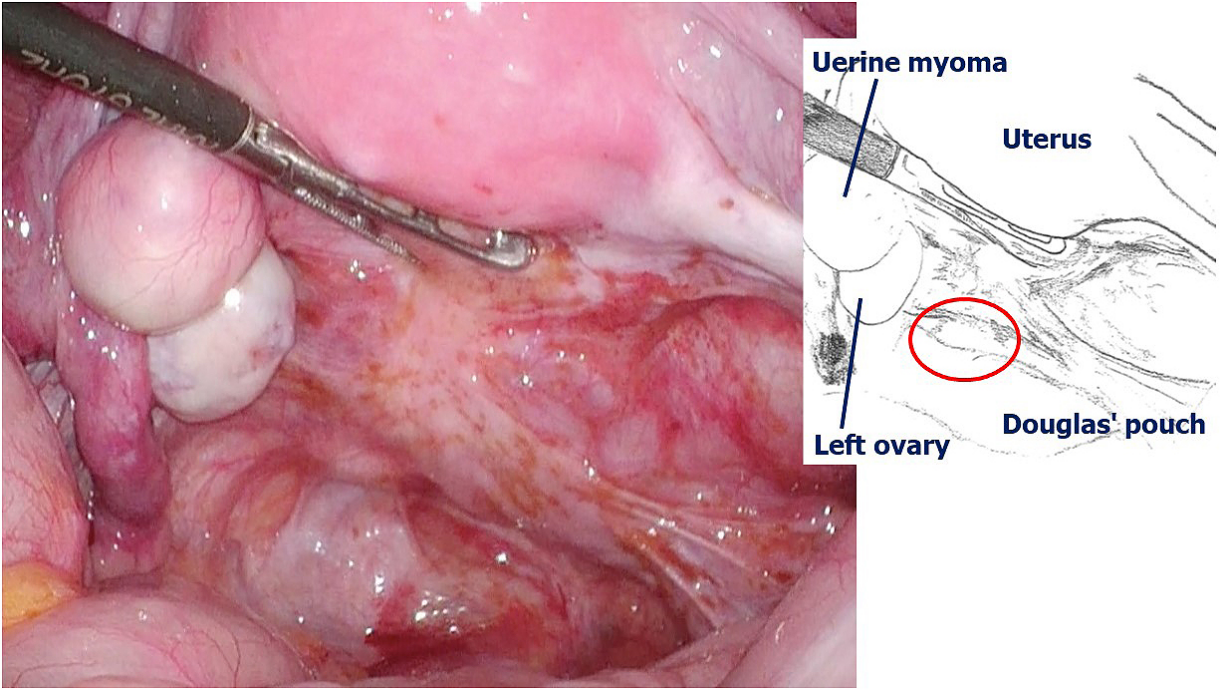
Intra-abdominal observations: absence of underlying conditions contributing to perforation (red circle: portion of perforation).

Intra-abdominal observations 3 months after the surgery confirmed the healing of the wound and absence of underlying conditions contributing to perforation, except endometriotic lesions scattered from the posterior surface of the uterus to the Douglas fossa (Figure 3).

## Discussion

In the current case, the intra-abdominal observations 3 months after the surgery revealed no problem in the sutured part of the vaginal wall and no underlying disease associated with the perforation. There were no large abnormalities in the abdominal cavity or vaginal wall.

Such a condition may be rare because we could not find similar cases after using the terms “cesarean section” with “vaginal wall perforation” or “perineal laceration” in Pubmed (https://pubmed.ncbi.nlm.nih.gov/) or Ichushi-Web of the Japan Medical Abstracts Society (https://www.jamas.or.jp/english/). However, this serious condition should be considered during cesarean delivery of the fetal head, as reported in the current case.

In this case, the cause of the perforation of the vaginal wall during the cesarean section remained unclear. Obstetricians should examine the vaginal wall carefully after lifting the fetal head during cesarean section. We did not perform a rough surgery in the subjective evaluation. In addition, we did not perform any transvaginal examination, such as lifting the fetal head during the cesarean section; however, we cannot deny the possibility that the vaginal wall was perforated due to the lifting of the fetal head. During the cesarean section, it is unlikely that a force is directly applied to the vaginal wall; however, some force may have been applied to the vaginal wall when the fetus was delivered. The perforation might also be attributed to the presence of tissue fragility due to endometriosis complicated by infection, such as in the current case, because endometriosis can cause the hardening of tissues around the vaginal wall ^(3)^. Especially in the cesarean section of primiparas at preterm, the vaginal wall may be hardened in preparation for delivery.

In conclusion, it will be necessary to carefully check the condition of the vagina even after the cesarean section. In addition, even if there are no abnormal findings in the examination of the vaginal wall immediately after the cesarean section, repeat examinations are recommended. It is also important to perform cesarean section in a protective manner.

## Article Information

### Conflicts of Interest

None

### Author Contributions

AW (primary author) analyzed the data and wrote the manuscript. SS conceptualized the study, analyzed the data, and drafted the manuscript. All authors critically reviewed the manuscript.

### Approval by Institutional Review Board (IRB)

IRB approval was not required for this study.

### Informed Consent

Written informed consent was obtained from the patient for the publication of the current report and patient images.
